# Multi-Axis Force/Torque Sensor Based on Simply-Supported Beam and Optoelectronics

**DOI:** 10.3390/s16111936

**Published:** 2016-11-17

**Authors:** Yohan Noh, Joao Bimbo, Sina Sareh, Helge Wurdemann, Jan Fraś, Damith Suresh Chathuranga, Hongbin Liu, James Housden, Kaspar Althoefer, Kawal Rhode

**Affiliations:** 1 Department of Informatics, King’s College London, London WC2R 2LS, UK; hongbin.liu@kcl.ac.uk; 2 Department of Biomedical Engineering, King’s College London, London SE1 7EH, UK; richard.housden@kcl.ac.uk (J.H.); kawal.rhode@kcl.ac.uk (K.R.); 3 Instituto Italiano di Tecnologia (IIT), Genova 16163, Italy; joao.bimbo@iit.it; 4 Department of Aeronautics, Imperial College London, London SW7 2AZ, UK; s.sareh@imperial.ac.uk; 5 Department of Mechanical Engineering, University College London, London WC1E 7JE, UK; h.wurdemann@ucl.ac.uk; 6 Industrial Research Institute for Automation and Measurements, Warsaw 02-486, Poland; jfras@piap.pl; 7 Department of Mechanical Engineering, University of Moratuwa, Katubadda, Moratuwa 10400, Sri Lanka; Chathurangas@uom.lk; 8 ARQ—Advanced Robotics @ Queen Mary Faculty of Science and Engineering Queen Mary University of London, Mile End Rd, London E1 4NS, UK; k.althoefer@qmul.ac.uk

**Keywords:** force/torque sensor, multi-axis force/torque, optoelectronic sensors, light intensity optics

## Abstract

This paper presents a multi-axis force/torque sensor based on simply-supported beam and optoelectronic technology. The sensor’s main advantages are: (1) Low power consumption; (2) low-level noise in comparison with conventional methods of force sensing (e.g., using strain gauges); (3) the ability to be embedded into different mechanical structures; (4) miniaturisation; (5) simple manufacture and customisation to fit a wide-range of robot systems; and (6) low-cost fabrication and assembly of sensor structure. For these reasons, the proposed multi-axis force/torque sensor can be used in a wide range of application areas including medical robotics, manufacturing, and areas involving human–robot interaction. This paper shows the application of our concept of a force/torque sensor to flexible continuum manipulators: A cylindrical MIS (Minimally Invasive Surgery) robot, and includes its design, fabrication, and evaluation tests.

## 1. Introduction

Robotic systems have developed considerably over the last ten years. New advances in actuators and materials allow the creation of more complex systems capable of conducting more advanced tasks than previously possible. Force and tactile sensing is one of the key research areas for robot systems, where such systems are required to characterise their interaction with the environment. With the ever-increasing demand to create robots that can safely interact with their environment, especially with humans, the need for robust, low-cost and miniature sensors is increasing too. It is well recognised that the acquisition of high-quality force and tactile sensor signals during a physical interaction between, say, a robot hand and an object being manipulated provides the opportunity to greatly improve the handling and manipulation capability of the robot system. Incorporating small-sized force/torque sensors in robot hands and arms has been shown to enhance obstacle avoidance, object grasping, in-hand manipulation and, generally, the meaningful interaction with the physical environment [1,2,3]. Current force measuring sensors show clear shortcomings due to their size, manufacturability, integration incompatibility, sensitivity, measurement range and/or lack of sufficient axes of measurement [4,5,6,7], with direct force measurements clearly outperforming approaches that use remote sensors to indirectly measure forces imparted on the robot structure. 

The force sensing approach proposed in this paper attempts to provide a solution to this issue whilst keeping costs low and representing an enabling technology for a wide range of application areas including medical robotics, manufacturing, and areas involving human–robot interaction. The main contributions of the presented work are:

- Integration of optoelectronic force sensors in a bespoke mechanical structure for the purpose of force/torque measurement;
- A concept that lends itself to miniaturisation;
- Simplified manufacturability and customisation to fit a wide-range of robot systems;
- Low-cost fabrication and assembly of sensor structure; and
- Good sensitivity and sensor range for a wide range of manipulation tasks.

A number of advanced robots have appeared recently in the research arena. Examples include dexterous, flexible snake-like manipulators that can potentially provide high dexterity and mobility in confined spaces that may not be easily achieved by traditional robotic systems [8,9,10,11,12]. However, most of these robots lack force-sensing capabilities—An aspect that becomes essential if one wants to operate a robot in an environment where physical interactions with surrounding objects are the norm. Hence, such robots cannot exploit their dexterity capabilities to the full, since interactions (collisions) with the environment remain largely undetected [13,14,15].

Using force sensors, robot hands can distinguish various kinds of tactile information including pressure and tangential forces, providing a sense of touch and enabling the equipped hands to perceive scratching, pricking, rubbing and the recognition of object shapes. The interpretation of the force sensor signals and, generally, the sense of touch have been extensively researched within the field of robotics [16,17,18,19]. 

One of the most advanced force/torque sensors is probably the six-axis sensor developed by DLR (German Aerospace Center) [20,21]. Despite its superior force/torque measurement capabilities, there are some shortcomings: (a) The sensor’s geometry cannot be easily adapted to a particular robot structure (because of the Stewart platform, which is the dominant feature of this sensor and cannot be altered without impacting on the measurement characteristics); (b) the sensor is relatively complex; and (c) expensive to manufacture (manually attachment of strain gauges on the sensor’s flexor beams). The superior measurement characteristics of the DLR force/torque sensor are acknowledged. Our paper is particularly interested in proposing a sensing concept that is low cost and can easily be integrated with a robotic structure—Hence, we do not see our sensor as a competitor to the DLR sensor with regards to measurement accuracy and sensitivity. 

Another important and widely used force/torque sensor is the ATI Nano17 sensor (ATI Industrial Automation, Apex, NC, USA)—This sensor is often seen as an industry standard for torque/force sensors, because of its outstanding sensing capabilities with regards to accuracy, sensitivity and range [22]. However, the sensor comes in a predefined housing (circular, 17 mm diameter, 14 mm length) which cannot be modified, and as such is of limited usefulness when further miniaturisation and adaptation to robot structures are required, although some researchers have succeeded in integrating these sensors with robot hands for improved interaction sensing. For example, during EU project HANDLE (Developmental pathway towards autonomy and dexterity in robot in-hand manipulation), the fingertips of the Shadow Robot Hand were equipped with Nano17 sensors to measure force and tactile information during robot-object interaction [23]. Good real-time measurements along all six axes were achievable, but the robot hand’s fingertips needed to be adapted and integrating the Nano17 sensors meant increasing the size of the fingertips; also the comparatively high weight of the sensors impacted on the fingers’ inertia and required control adjustments. It is also noted that an ATI sensor is a relatively expensive item. In this paper, the ATI sensor is used for the calibration and evaluation of our sensor (see Section 3).

**Figure 1:**
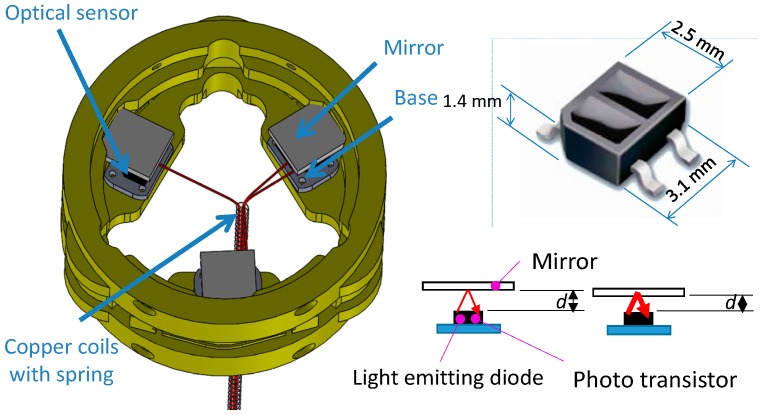
Assembly design for three axis force/torque sensor using three optoelectronic sensors and three mirrors, optoelectronic sensor, QRE1113, Fairchild Semiconductor Corp. (South Portland, ME, USA).

**Figure 2:**
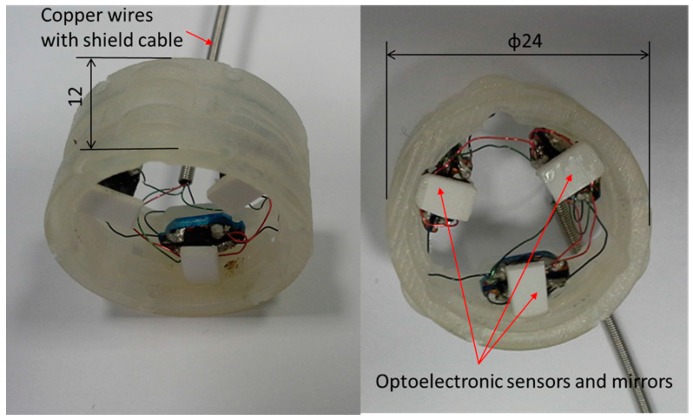
Fabricated three-axis force/torque sensor.

**Figure 3:**
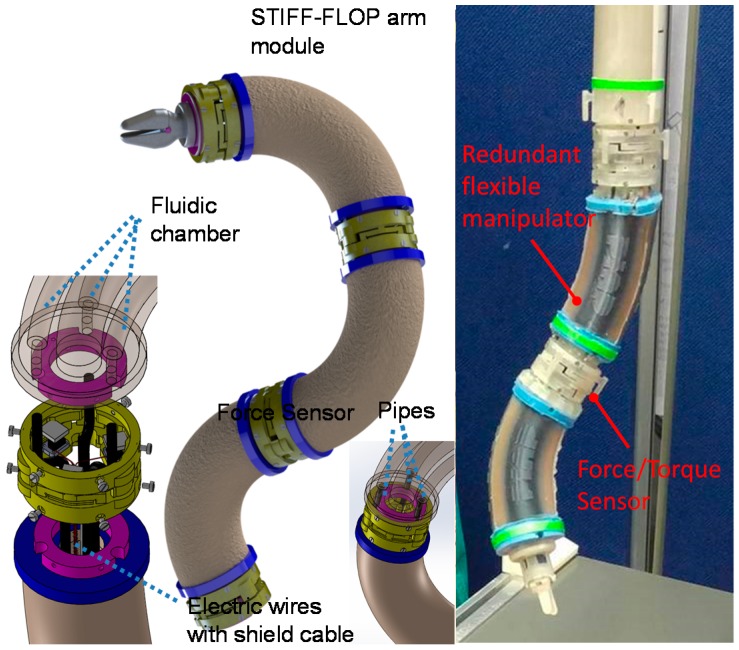
Flexible continuum manipulators: STIFF-FLOP arm with force/torque sensors.

In general, most available force sensors are fabricated without the structure that they will be used on in mind. This complicates their integration with the intended structure. This becomes particularly problematic in cases where the robot structure is small, as is often the case with manipulation devices such as robot hands and robots used for minimally invasive surgery [4,5,24]. In such cases, having the capability of adapting the sensor geometry to the robot’s structure is essential. In some cases, there is additionally the need of extending the structural geometry of the robot at the point where the sensor is to be integrated. A number of robots or, in some cases, their links can be represented by hollow, cylindrical structures where the inner space is used to feed through auxiliary cables, tubes and/or tendons. Integrating a “conventional” sensor (which commonly does not provide empty space internally) is not possibly in such a case without significantly modifying the overall robot structure [13,14]. Here, our sensor concept excels because it provides the required design freedom to align the sensor structure with the robot structure. In our chosen example, where the force/torque sensor is to be integrated with the STIFF-FLOP (STIFFness controllable Flexible and Learn-able Manipulator for surgical OPerations) arm [25,26,27,28,29,30], the sensor structure is designed to be ring-shaped and, thus, easily integrated between robot segments, allowing multiple cables and tubes to be passed through (Figure 1, Figure 2 and Figure 3).

In general, our work aims at creating force/torque sensors that are most suitable for integration with a range of robot systems, particularly snake-like and highly redundant arms to measure the interaction of their individual links with the environment and robot hands to measure forces during grasping and manipulation events. Particular emphasis is on the creation of low-cost, easily manufacturable and integratable sensors for the real-time measurement of force and torque signals. With a particular interest in medical robotics, we will demonstrate the integration of our sensor with a continuum robot (the STIFF-FLOP arm), which is intended for use during minimally invasive surgery. Having such sensors integrated with these types of robots, we can provide physical interaction information such as force and tactile perception in surgical environments during an operation. Having this information readily available, compliant force control can be incorporated and used to move complex robot structures whilst preventing excessive forces from being applied to the clinical environment [31,32]. 

**Figure 4:**
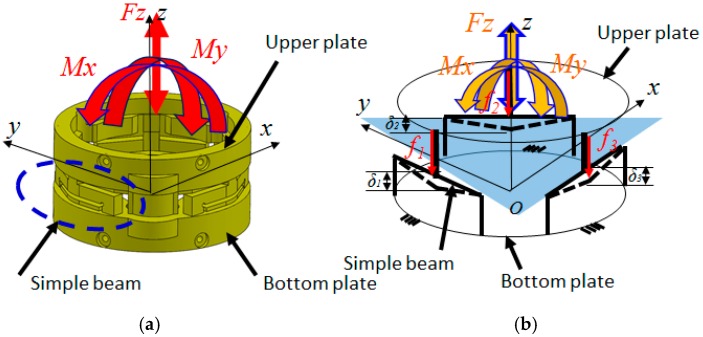
Measurable force and moment components on (**a**) the force/torque sensor and (**b**) simplified sensor structure model (of simply-supported beam).

Although there are many methods to measure deformation to calculate external force/torque, for example by attaching polyvinylidene fluoride (PVDF) films [33], strain gauges [20], piezoresistive materials [34], or fibre bragg grating (FBG) [21,35] on the sensor structures, we are proposing light intensity-based measurement using optoelectric technology [36,37,38] and simply-supported beam (Figure 1 and Figure 2) [39]. Our proposed methods have advantages such as immunity to electrical noise, low power consumption, low-level noise, no need for electronic filtering, easy attachment into the sensor body, and low cost [40,41,42,43,44,45]. In addition, recent technological advances allow diminutive sized photo sensor (1.0 × 1.4 × 0.6 mm^3^) [46]. To calculate force/torque components, optoelectronic sensors are deployed along the circumference of the sensor structure and can measure three deflections (*δ*_1_, *δ*_2_ and *δ*_3_) (Figure 1, Figure 2 and Figure 4). This simplifies manufacturing, makes the overall sensor size miniaturised, and secures an ample space in the centre of the sensor structure. This is in contrast with conventional methods as mentioned above, so it is hard to secure an ample space in the centre of the sensor structure.

Furthermore, today’s 3D printing technologies allow low cost fabrication of miniaturised complex sensor structures in metal. Hence, the overall size of the sensors can be much smaller than commercially available force/torque sensors. In this paper, the detailed design and calibration of a proposed multi-axis force/torque sensor with a ring-like structure and hollow inner section providing ample space for auxiliary components is presented. Employing the STIFF-FLOP manipulator as an example, we show how our sensor can be customised to fit a particular robot structure. The sensor is calibrated and, hence, through an experimental study, we obtain and validate its calibration matrix. This study also evaluates various properties such as measurement error, repeatability, hysteresis and crosstalk.

## 2. Design Methods and Fabrication

This section will describe the design requirements, the configuration and the analysis for an optimized force/torque sensor, specifically for integration with the STIFF-FLOP robot arm design.

### 2.1. Design Requirements

The design of the force/torque sensor should satisfy the following requirements:

(1) Be capable of measuring forces and moments in a range suitable for the application: In [47,48,49], the force applied by robotic surgical systems ranges from 0 to 21 N.
(2) Be miniaturisable: The diameter of sensor structure should be less than 15 mm to be able to pass through the current commercialized trocars.
(3) Be easily adaptable and integrateable with the given manipulator structure.
(4) Impact on the robot structure as little as possible and provide space for necessary tubes and cables for other functions such as robot actuation, sensing, and tool actuation.

In this paper, to prove the efficiency of our proposed three-axis force sensor for being integrated into the STIFF-FLOP manipulator (Diameter: 25 mm, Length: 50 mm, four 1 mm or 2 mm pipes are required for one manipulator as shown in Figure 3), we designed the first prototype with the measurement range and size described in Table 1.

### 2.2. Configuration of Optoelectronic Based Force/Torque Sensor

The structure of the optoelectronic force/torque sensor is shown in Figure 1 and Figure 2. The force/torque sensor uses an optoelectronic sensor, QRE1113 from Fairchild Semiconductor Corp. (Figure 1), with reflective plastic as a mirror [50]. A flexible ring-like structure made of ABS plastic (Visijet^®^ EX200, 3D Systems Corporation, Rock Hill, SC, USA, a copolymer of Acrylonitrile, Butadiene, and Styrene) using rapid prototyping (Project^TM^ HD-3000 Plus, 3D Systems Corporation, Rock Hill, SC, USA) was created to measure one force component *F_z_*, and two moment components, *M_x_* and *M_y_*, as illustrated in Figure 4. In order to measure the three components of force and moments, the three deflections *δ*_1_, *δ*_2_ and *δ*_3_ in the sensor structure based on the simply-supported beam need to be measured through three optoelectronic sensors (Figure 4).

Each of our individual force sensing elements makes use of a pair of optoelectronics consisting of a light-emitting diode and a phototransistor. The optoelectronics are appropriately combined with a reflective surface (mirror) to reflect the light emitted by the light-emitting diode then received by the phototransistor (Figure 1 and Figure 2). Any variation in distance with regards to the optoelectronics due to an applied force changes the amount of light reflected onto the phototransistor. The light received by the phototransistor element is converted into an analogue voltage signal and subsequently converted into a digital signal for further processing on a computer. 

In the event that an external force and moment *F_z_*, *M_x_* and *M_y_* are applied to the upper plate of the sensing structure, the three associated beams are deflected. The three corresponding optoelectronic sensing elements measure the resultant beam deflections (*δ*_1_, *δ*_2_ and *δ*_3_) between the upper plate and bottom plate (Figure 4). The deflections *δ*_1_, *δ*_2_ and *δ*_3_ can be determined from the read-out output voltages of the receiving elements of the optoelectronics, i.e., the photo transistors.

**Figure 5:**
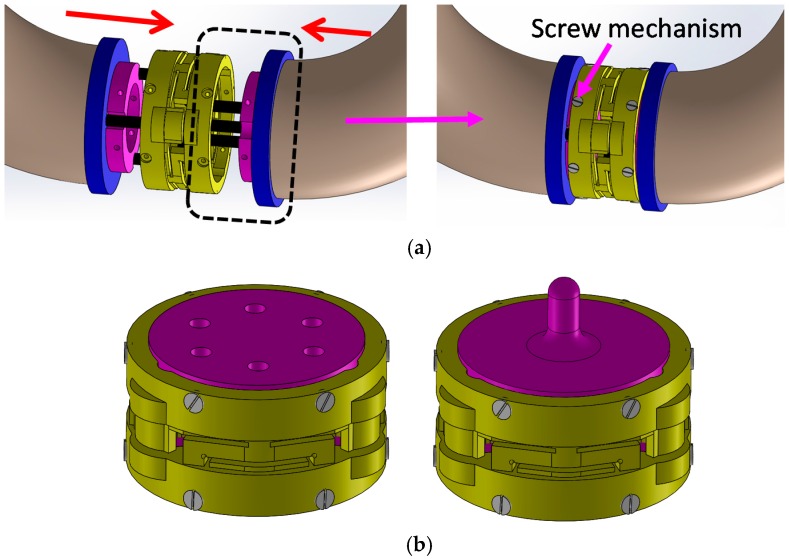
Screw mechanism of three-axis force torque sensor: (**a**) The STIFF-FLOP manipulator with screw mechanism; (**b**) Three-axis force/torque sensor (**left**) and three-axis force sensor (**right**) with screw mechanism.

In addition, in order to anchor the force sensor to the structure of the STIFF-FLOP arm, 12 screws are used, as shown in Figure 4 and Figure 5. The hollow structure of our force sensor allows pipes and electrical wires to pass through. By modifying the shape of the bottom and top connection parts (purple components), the proposed sensor can be easily adapted to a wide range of robot structures (Figure 5).

### 2.3. Sensor Structure Design, Modelling, Simulation, and Sensor’s Optoelectronic Optimization

**Figure 6:**
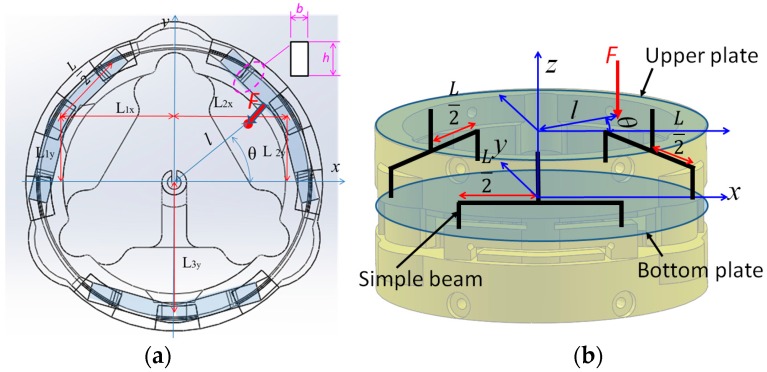
Sensor structure (section of simply-supported beam) dimension and design variables (L = 6 mm, *L*_1*x*_ = 9.3 mm, *L*_1*y*_ = 5.4 mm, *L*_2*x*_ = 9.3 mm, *L*_2*y*_ = 5.4 mm, *L*_3*y*_ = 10.8 mm, *h* = 1.5 mm, and *b* = 1.5 mm): (**a**) The top view; (**b**) The side view.

To measure the force component and the two moments, the structure of the ring-shaped force/torque sensor can be simplified to three simply-supported beams arranged within the sensor structure, with an angular distance of 120° between them, as shown in Figure 1 and Figure 6. In the event that an external force is applied on the upper plate, each of the three simply-supported beams is deflected. The three photo sensors can measure the amount of deflection *δ*_1_, *δ*_2_ and *δ*_3_, and from the three deflections, the two moments *M_x_*, *M_y_* and force *F_z_* can be measured in terms of *f*_1_, *f*_2_ and *f*_3_:

(1) $$f_{1}=k_{1}\delta_{1}$$

(2) $$f_{2}=k_{2}\delta_{2}$$

(3) $$f_{3}=k_{3}\delta_{3}$$

Exploiting knowledge of the beam material properties, the deflections of the simply-supported beams are calculated and spring coefficient *k* can be obtained [39]:

(4) $$I=\frac{bh^{3}}{12}$$

(5) $$\delta =\frac{fL^{3}}{48EI}$$

(6) $$k=\frac{48EI}{L^{3}}$$

where spring coefficient *k* is modelled as a function of the beam length *L*/2, the modulus of elasticity *E*, the moment of inertia *I*, the width of beam’s section *b*, and the beam’s section *h* as shown in Figure 6a. 

In the event that an amount of external force is applied to the upper plate (as shown in Figure 6), force *F_z_* and moment components *M_x_* and *M_y_* can be calculated by substituting all of the variables as follows:

(7) $$F_{z}=f_{1}+f_{2}+f_{3}=k\delta_{1}+\;k\delta_{2}+k\delta_{3}$$

(8) $$M_{x}=F\cdot l\cdot sin\theta =-L_{1y}\cdot f_{1}-L_{2y}\cdot f_{2}+L_{3y}\cdot f_{3}$$

(9) $$M_{y}=F\cdot l\cdot cos\theta =-L_{1x}\cdot f_{1}+L_{2x}\cdot f_{2}$$

The deflections of the three simply-supported beams are represented, in turn, by the output voltages (*v*_1_, *v*_2_ and *v*_3_) of the three optoelectronic sensing elements, as follows:

(10) $$f_{1}=k\delta_{1}=m_{1}v_{1}$$

(11) $$f_{2}=k\delta_{2}=m_{2}v_{2}$$

(12) $$f_{3}=k\delta_{3}=m_{3}v_{3}$$

where constants *m*_1_, *m*_2_ and *m*_3_ are obtained experimentally. From Equations (10) to (12), the external force *F_z_* and moment components *M_x_* and *M_y_* can be calculated by measuring *f*_1_, *f*_2_ and *f*_3_ as shown in Figure 4 (right). The calibration matrix ***k_v_*** can be obtained as follows:

(13) $$\left[\begin{array}{c} F_{z}\\ M_{x}\\ M_{y} \end{array}\right]=k_{v}\cdot v=\left[\begin{array}{ccc} k_{v11} & k_{v12} & k_{v13}\\ k_{v21} & k_{v22} & k_{v23}\\ k_{v31} & k_{v32} & k_{v33} \end{array}\right]\left[\begin{array}{c} v_{1}\\ v_{2}\\ v_{3} \end{array}\right]=\left[\begin{array}{ccc} m_{1} & m_{2} & m_{3}\\ -m_{1}L_{1y} & -m_{2}L_{2y} & m_{3}L_{3y}\\ -m_{1}L_{1x} & -m_{2}L_{2y} & 0 \end{array}\right]\left[\begin{array}{c} v_{1}\\ v_{2}\\ v_{3} \end{array}\right]$$

#### 2.3.1. Optimization of Characteristic Curves on Optoelectronic Sensor

**Figure 7:**
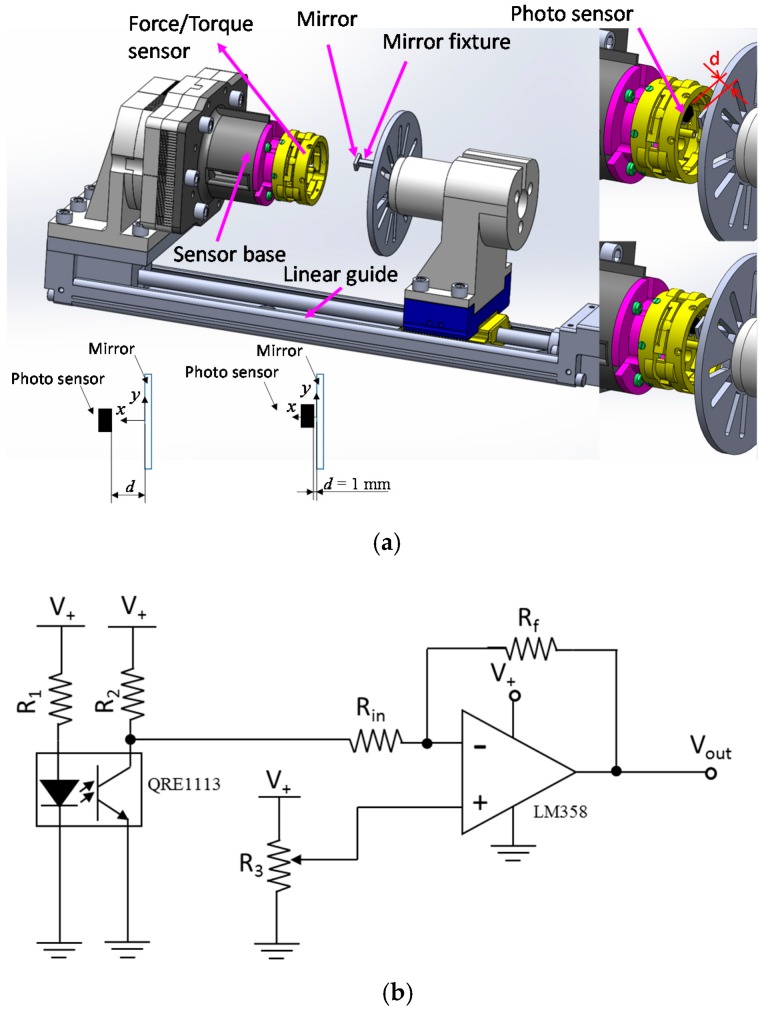
(**a**) Experimental setup to find optimized distance between an optoelectronic sensor and a mirror; (**b**) The inverting amplifier circuit with an optical sensor (R_1_ = 100 Ω, R_2_ = 5 kΩ, R_3_ = variable resistor for offset, R_in_ = 100 Ω, and R_f_ = 9 kΩ).

For the development of the three-axis force sensor, first of all, the relationship between deflections (*δ*_1_, *δ*_2_ and *δ*_3_) and output voltages of the optoelectronic sensors (*v*_1_, *v*_2_ and *v*_3_) need to be obtained. As shown in Figure 7, the calibration system consists of a linear guide, a sensor base, and a mirror. The mirror was set at the initial position where the distance between mirror and optoelectronic sensor was 1 mm, and moved 9 mm backward such that distance *d* from the optoelectronic sensors was increased. 

**Figure 8:**
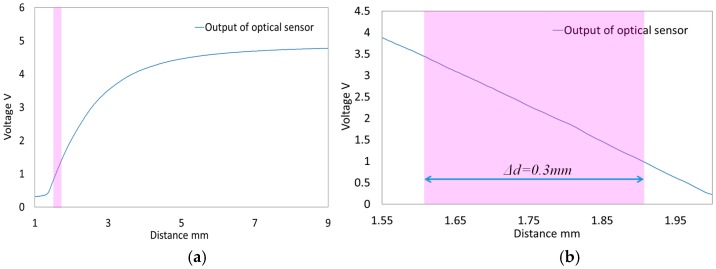
Characteristic curve of output voltage of optoelectronic sensor according to changing d: (**a**) Without amplifier; (**b**) With amplifier.

The outcome from our experimental study is the characteristic curves relating the output voltages of the optoelectronics (*v*_1_, *v*_2_ and *v*_3_) to distance *d* (Figure 8a). For the development of the force/torque sensor, the following requirements should be satisfied: (1) Small deflections yield large changes in voltage; (2) linearity. From the characteristic curves shown in the purple area in Figure 8a, the characteristic curves are fairly linear, but with small voltage changes for small changes in distance.

To obtain large voltage change with respect to small distance changes, an inverting amplifier was used as shown in Figure 7b and Figure 8b. The output shows linear behaviour in the region highlighted in purple, with *∆d* = 0.3 mm and a 2.5 V voltage change. For this reason, when force *F_z_* and moments *M_x_* and *M_y_* are applied to the upper plate as shown in Figure 4 and Figure 6, each of the three beam deflections should not exceeded *δ* = ±0.15 mm because the sensor output shows a linear behaviour within the distance *Δd* = 0.3 mm, as shown in Figure 8b. In addition, to measure positive or negative deflection when force and moment components are applied on the sensor structure, the initial output voltage of the optoelectronic sensor should be in the middle of the overall voltage range, i.e., here, at *d* = 1.75 mm in the purple area of Figure 8b.

#### 2.3.2. Model and Simulation for Sensor Structure

Depending on the desired measurement range of the force and moments, the sensor structure can be customized by choosing design parameters *L*, *E*, *I*, *b* and *h* as depicted in Figure 6 and in Equations (4)–(6). In this paper, to prove the efficiency of our proposed three-axis force sensor for the STIFF-FLOP arm, we designed the first prototype with a size and measurement range as shown in Table 1.

**Figure 9:**
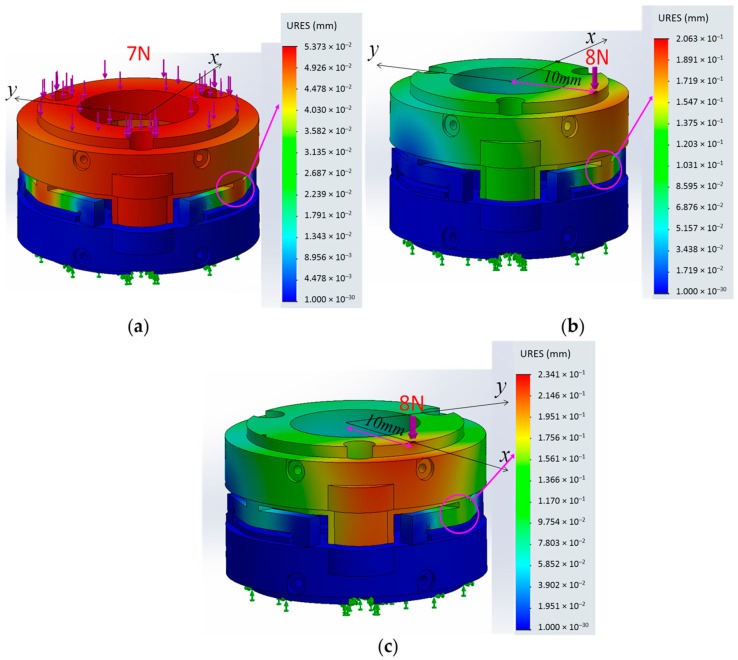
The FEM Simulation (the simulations performed using Solidworks FEM Simulation tool): (**a**) when *F_z_* is applied on the upper plate, the simply-supported beam deflects around *δ =* 0.05 mm; (**b**) when *F_z_* and *M_x_* are applied on the upper plate, the simply-supported beam deflects around *δ =* 0.15 mm; and (**c**) when *F_z_* and *M_y_* are applied on the upper plate, the simply-supported beam deflects around *δ =* 0.15 mm.

**Figure 10:**
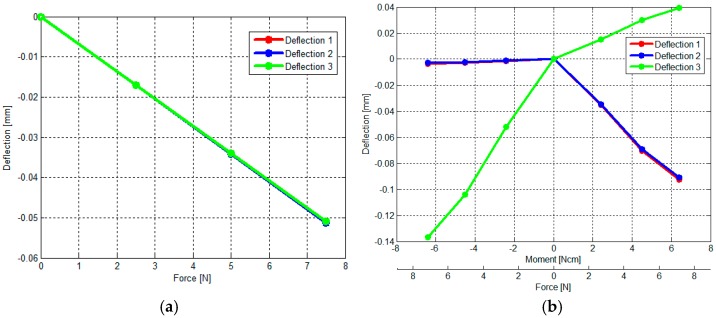

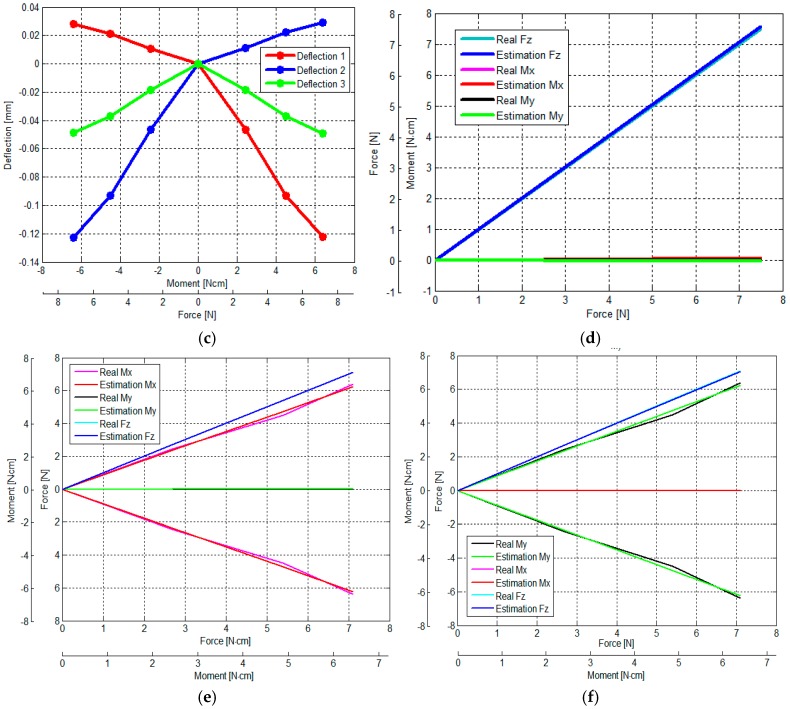
The simulation results describing simply-supported beam deflections and sensor’s accuracy and crosstalk evaluation: (**a**) when *F_z_* is applied on the upper plate, the simply-supported beam deflects around *δ* = 0.05 mm (see also Figure 9a); (**b**) when Force *F_z_* and Moment *M_x_* are applied on the upper plate, the simply-supported beam deflects around *δ* = 0.15 mm as shown (see also Figure 9b); (**c**) when Force *F_z_* and Moment *M_y_* are applied on the upper plate, the simply-supported beam deflects around *δ* = 0.15 mm (see also Figure 9c); (**d**) when *F_z_* is applied on the upper plate maximum error *F_z_* is around 1% and crosstalk *M_x_* and *M_y_* are less than 0.5% (the sensor accuracy and crosstalk simulated by FEM simulation) (see also Figure 9a); (**e**) similarly when Force *F_z_* and Moment *M_x_* are applied on the upper plate, maximum error *M_x_* is around 3% and crosstalk *M_y_* is less than 0.5% (see also Figure 9b); and (**f**) when Force *F_z_* and Moment *M_y_* are applied on the upper plate, maximum error *M_y_* is around 3% and crosstalk *M_x_* is less than 0.5% (see also Figure 9c).

In our simulations, the values associated with the material properties for the 3D-printed beams as well as the remainder of the sensor structure were set based on the information provided by the 3D-printer manufacturer (PROJET VisiJet^®^ EX200, 3D Systems Corporation), as follows: tensile modulus of 1283 MPa, mass density of 1020 kg/m^3^, and tensile strength of 42.5 MN/m^2^. In this simulation, we verified whether the sensor structure based on the three simply-supported beams could measure a certain range of force and moment components as summarized in Table 1. The simulation of exertion of force on the sensor structure implies a linear relationship between force and moment values and the deflections of the three beams (within the measurable ranges: −0.15 < δ < +0.15) (Figure 9 and Figure 10a–c). 

The Multiple Linear Regression attempts to model the relationship between two or more independent variables and a dependent variable, by fitting a linear equation to the observed data [51]. In this implementation, every value of an independent variable (the deflections of the three beam) is associated with a value of the dependent variables, force *F_z_* and moments *M_x_* and *M_y_*. Applying Multiple Linear Regression with independent variables *δ*_1_, *δ*_2_ and *δ*_3_ as shown in Figure 10a–c, the calibration matrix was calculated for *F_z_*, *M_x_* and *M_y_*. As shown in Equations (14) and (15), from the deflections of the three beams *δ*_1_, *δ*_2_ and *δ*_3_, the estimated *F_z_*, *M_x_* and *M_y_* can be obtained through the calculated calibration matrix.

Therefore, the force and moment components were estimated by multiplying the calibration matrix and the deflections of the three beam as shown in Figure 10d–f) and sensor’s performance parameters were calculated (accuracy (3%) and crosstalk (0.5%)). 

(14) $$\left[\begin{array}{c} Calibration\\ Matrix \end{array}\right]\left[\begin{array}{c} Three\\ Beam\\ Deflections \end{array}\right]=\left[\begin{array}{c} Force\\ and\\ Moments \end{array}\right]$$

(15) $$\left[\begin{array}{ccc} k_{\delta 11} & k_{\delta 12} & k_{\delta 13}\\ k_{\delta 21} & k_{\delta 22} & k_{\delta 23}\\ k_{\delta 31} & k_{\delta 32} & k_{\delta 33} \end{array}\right]\left[\begin{array}{c} \delta_{1}\\ \delta_{2}\\ \delta_{3} \end{array}\right]=\left[\begin{array}{c} F_{z}\\ M_{x}\\ M_{y} \end{array}\right]$$

## 3. Sensor Calibration

### 3.1. Setup for Calibration Experiments

**Figure 11:**
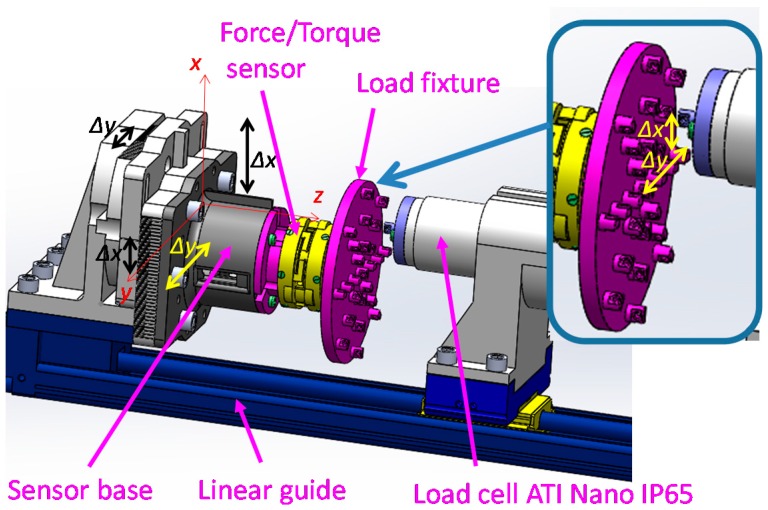
Assembly design of sensor calibration device.

**Figure 12:**
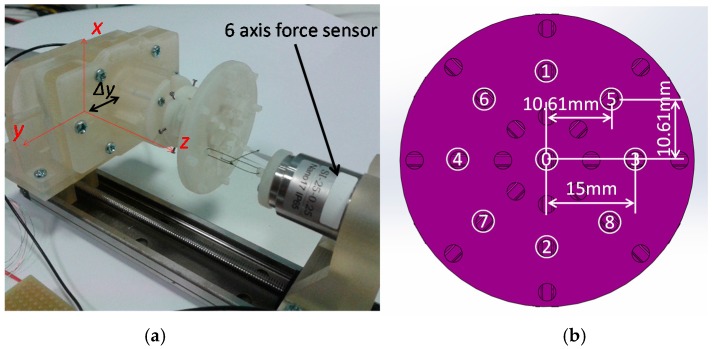
Sensor calibration device: it can apply compressive force by a load tip and tensile force by a wire, at the numbered points on the load fixture. (**a**) Sensor calibration device; (**b**) Detail description of the load fixture as shown in Figure 11.

Sensor calibration is the process of finding the relationship between a physical value and the output voltage of the sensor. In our application, the physical values are force and moment components: *F_z_*, *M_x_* and *M_y_*. In order to find the relationship between the force and moment components and the output voltages of the three optoelectronic sensing elements, a calibration device is proposed as shown in Figure 11 and Figure 12. This device consists of a linear guide, a sensor base, a load fixture and a load cell (ATI Nano IP65). The force/torque sensor and the load fixture are mounted on the sensor base (Figure 11 and Figure 12). The sensor base can move along the *x*-axis and *y*-axis to investigate the three following conditions:

(1) Force *F_z_* along *z*-axis as depicted ⓪ (in Figure 12b);
(2) Force *F_z_* and moment *M_x_* or *M_y_* (denoted as (*F_z_*, *M_x_*) ①② or (*F_z_*, *M_y_*) ③④ (in Figure 12b)); and
(3) Force *F_z_* and both moments *M_x_* and *M_y_* (denoted as (*F_z_*, *M_x_*, *M_y_*) ⑤⑥⑦⑧ (in Figure 12b)) while an actuator moves along the *z*-axis as shown in Figure 11 and Figure 12.

**Figure 13:**
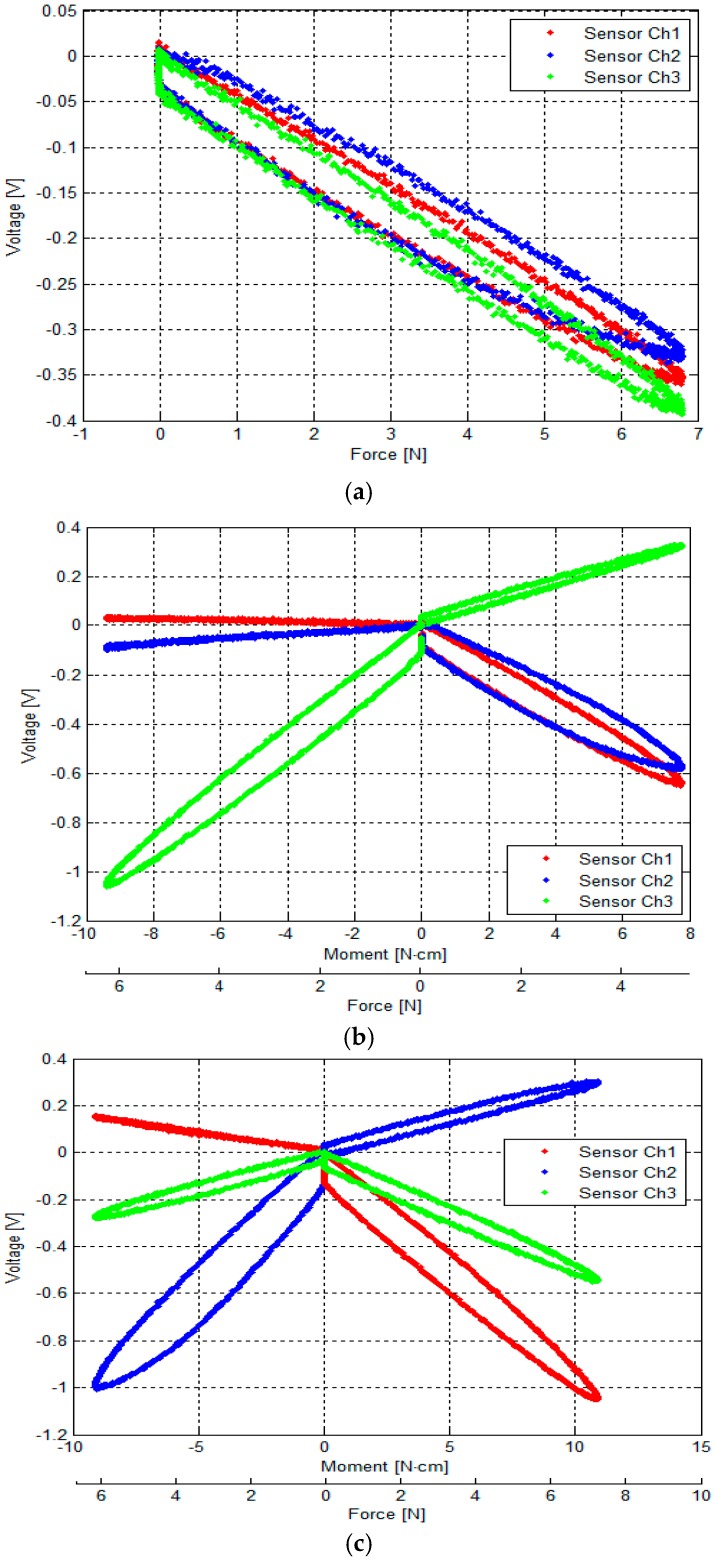
Characteristic curves between the physical loads (force/moment) and the output voltage of the force sensor: (**a**) Between the load (*F_z_*) and the output voltages of the force sensor: external force was applied to and released from point ⓪ by the sensor calibration device as shown in Figure 12b; (**b**) between the loads (*F_z_*, *M_x_*) and the output voltage of the force sensor: external force was applied to and released from points ① and ②; and (**c**) between the loads (*F_z_*, *M_y_*) and the output voltage of the force sensor: External force was applied to and released from points ③ and ④.

In these experiments, a tensile force and a moment are applied to find the relationship between physical force/moment from the load cell and the output voltages of the three optoelectronic sensing elements of our sensor. The moment is calculated by multiplying the tensile force and a distance as shown in Figure 12. The experimental results show the sensor structure has large hysteresis due to the use of ABS plastic materials (Figure 13) while loading and unloading force and moment values are consistent with the simulation results (Figure 10a–c).

### 3.2. Calculation of Calibration Matrix by Analytical and Empirical Approaches

Sensor calibration was carried out using the calibration device, examining these three conditions. The results of the calibration are shown in Figure 13:

- Figure 13a shows the relationship between the output voltages of the three optoelectronic sensing elements and *F_z_*.
- Figure 13b shows the relationship between output voltages of the three optoelectronic sensing elements and (*F_z_*, *M_x_*).
- Figure 13c shows the relationship between the output voltages of the three optoelectronic sensing elements and (*F_z_*, *M_y_*).

All graphs show that the relationships between the physical values, *F_z_*, (*F_z_*, *M_x_*), and (*F_z_*, *M_y_*), and the corresponding sensor output voltages are relatively linear, albeit suffering from noticeable hysteresis. It is noted that there is a degree of cross coupling between the sensing elements, i.e., the load on one axis produces a change in output on other axes. Here, each optoelectronic sensing element is individually calibrated with loads applied along each axis. 

The calibration data are used to generate a calibration matrix, which is used to convert the output voltages to force and moment loading data. The three-by-three calibration matrix is multiplied by the three-element voltage vector (column) to obtain the calibrated, decoupled output (Equation (13)):

(16) $$k_{v}=\left[\begin{array}{ccc} -6.3846 & -6.8163 & -5.9703\\ -3.4476 & -3.6808 & 6.4479\\ -5.9376 & 6.3391 & 0 \end{array}\right]$$

(17) $$\left[\begin{array}{c} Calibration\\ Matrix \end{array}\right]\left[\begin{array}{c} Sensor\\ Output\\ Volts \end{array}\right]=\left[\begin{array}{c} Force\\ and\\ Moments \end{array}\right]$$

(18) $$\left[\begin{array}{ccc} k_{e11} & k_{e12} & k_{e13}\\ k_{e21} & k_{e22} & k_{e23}\\ k_{e31} & k_{e32} & k_{e33} \end{array}\right]\left[\begin{array}{c} v_{1}\\ v_{2}\\ v_{3} \end{array}\right]=\left[\begin{array}{c} F_{z}\\ M_{x}\\ M_{y} \end{array}\right]$$

(19) $$k_{e}=\left[\begin{array}{ccc} -5.6456 & -5.3600 & -5.5298\\ -5.1774 & -2.9146 & 8.5146\\ -7.4585 & 7.8664 & -0.7881 \end{array}\right]$$

From the experiment result as shown in Figure 13a, the three constants (*m*_1_, *m*_2_ and *m*_3_) are obtained experimentally, the analytical calibration matrix *k_v_* is calculated by Equation (13) and parameters (Table 1) as shown in Equation (16). At the same time, the calibration matrix *k_e_* is also calculated by empirical approach, Multiple Linear Regression.

Applying Multiple Linear Regression with independent variables *v*_1_, *v*_2_ and *v*_3_ as shown in Figure 13, the calibration matrix was calculated for *F_z_*, *M_x_* and *M_y_*. As shown in Equations (17)–(19), from the sensor voltage samples *v*_1_, *v*_2_ and *v*_3_, the estimated *F_z_*, *M_x_* and *M_y_* can be obtained through the calculated calibration matrix.

Although the analytically calculated calibration matrix (Equation (16)) is not exactly the same as the empirically obtained one (Equation (19)), they are considerably analogous. 

### 3.3. Verification of Calibration Matrix and Objective Evaluation of Our Proposed Force/Torque Sensor 

In order to evaluate the proposed three-axis force/torque sensor objectively, sensor properties such as crosstalk, hysteresis, repeatability, and error should be examined. For these reasons, a set of experiments was carried out using the calibration device shown in Figure 11 and Figure 12. In order to verify our calibration matrix and evaluate the sensor properties, the loading/unloading calibration process was performed. The calibration considered the three conditions of the benchmark forces at the nine points, as explained in Section 3.1, and was performed five times at a linear guide speed of 0.15 mm/s consecutively.

(1) Force *F_z_* along *z*-axis ⓪ (in Figure 12b);
(2) Force *F_z_* and moment *M_x_* or *M_y_* (from now express as (*F_z_*, *M_x_*) ①② or (*F_z_*, *M_y_*) ③④) (in Figure 12b); and
(3) Force *F_z_* and both moments *M_x_* and *M_y_* (from now express as (*F_z_*, *M_x_*, *M_y_*) ⑤⑥⑦⑧) (in Figure 12b).

**Figure 14:**
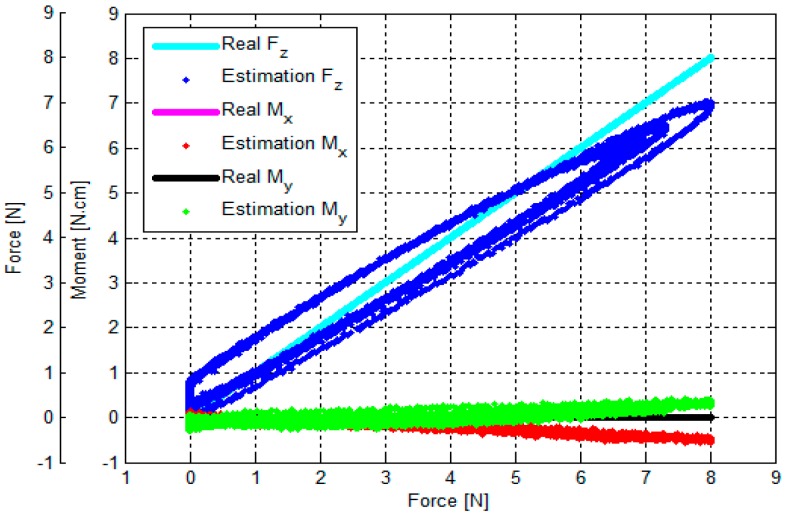
Comparison between real and estimated *F_z_* values: an external force was applied to and released from point ⓪ by the sensor calibration device as shown in Figure 12 (note that the values of *M_x_* and *M_y_* overlap, being both zero at all times).

**Figure 15:**
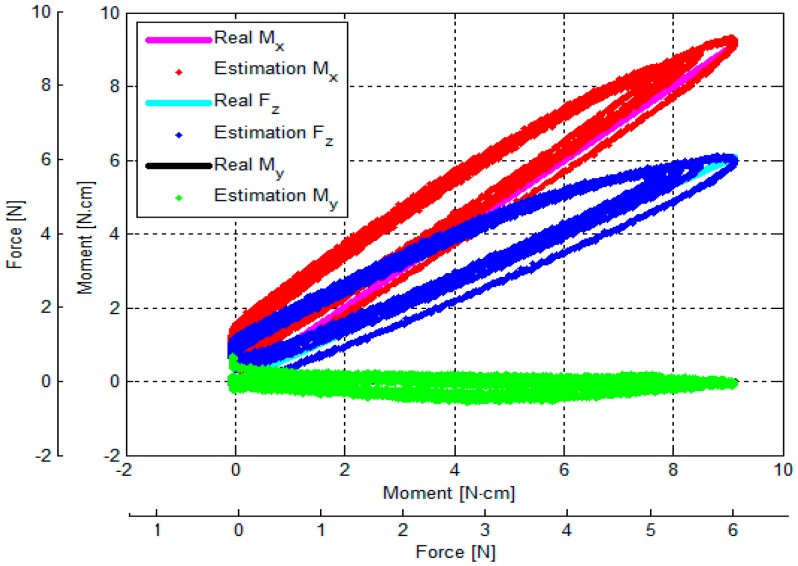
Comparison between real (*F_z_*, *M_x_*) and estimated (*F_z_*, *M_x_*): external force was applied to and released from point ① obtained by the sensor calibration device as shown in Figure 12.

**Figure 16:**
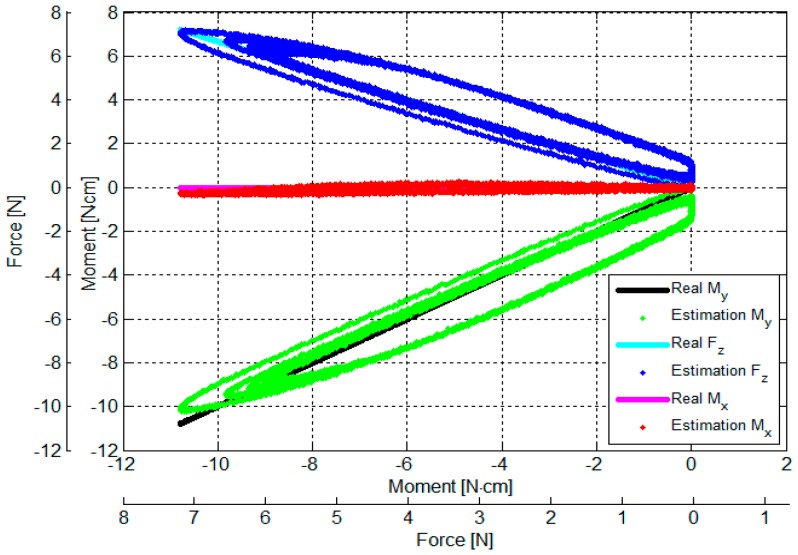
Comparison between real (*F_z_*, *M_y_*) values and estimated (*F_z_*, *M_y_*): external force was applied to and released from point ③ obtained by the sensor calibration device as shown in Figure 12.

**Figure 17:**
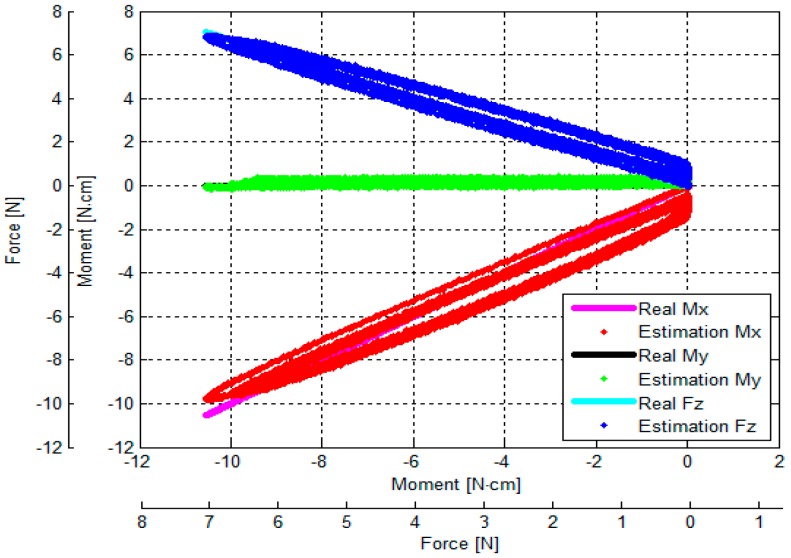
Comparison between real (*F_z_*, *M_x_*) and estimated (*F_z_*, *M_x_*): external force was applied to and released from point ② obtained by the sensor calibration device as shown in Figure 12.

**Figure 18:**
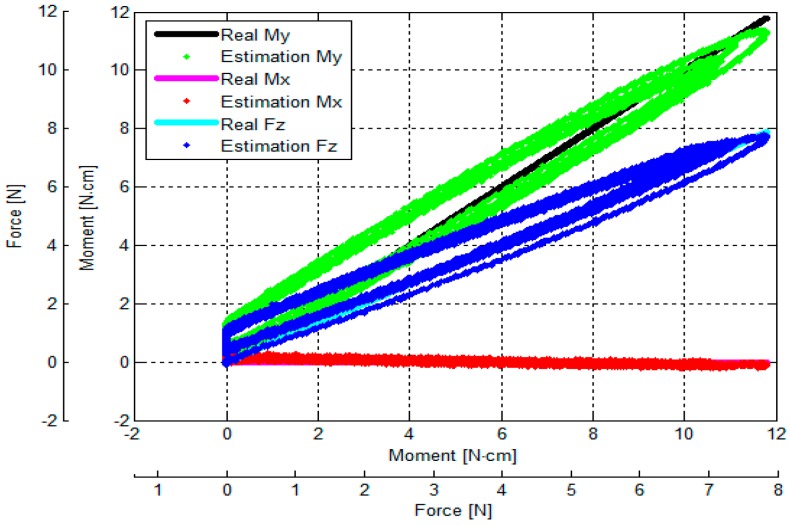
Comparison between real (*F_z_, M_y_*) values and estimated (*F_z_, M_y_*): external force was applied to and released from point ④ obtained by the sensor calibration device as shown in Figure 12.

**Figure 19:**
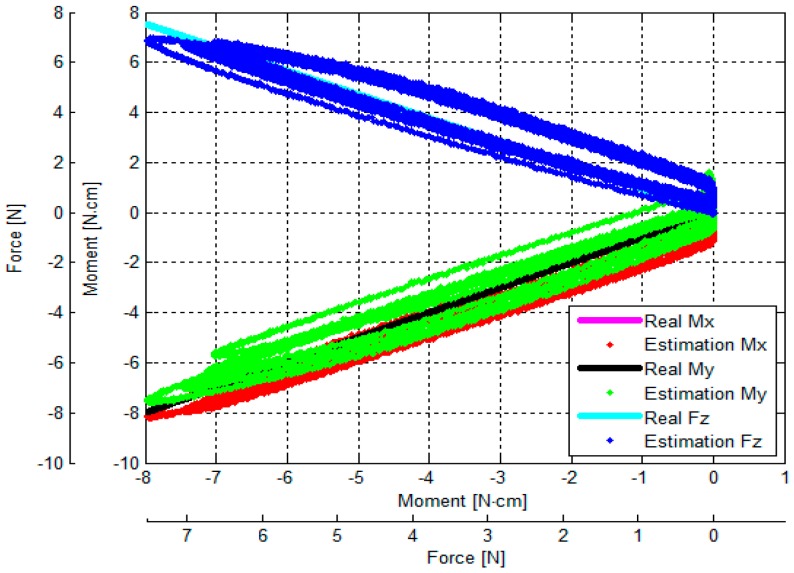
Comparison between real (*F_z_*, *M_x_, M_y_*) values and estimated (*F_z_*, *M_x_, M_y_*): external force was applied to and released from point ⑧ obtained by the sensor calibration device as shown in Figure 12.

The output voltages of the three optoelectronic sensing elements during the calibration process were recorded. Subsequently, estimated values of *F_z_*, *M_x_* and *M_y_* were calculated by multiplying values of the voltage samples and the calibration matrix, presented in Equation (18). Overall, based on the experimental results, the *F_z_*, *M_x_* and *M_y_* calculated by the calibration matrix are in agreement with the corresponding force values as measured by the ATI Nano17 IP65 force/torque sensor, as shown in Figure 14, Figure 15, Figure 16, Figure 17, Figure 18 and Figure 19. 

As the cycle numbers increase, the knot of the two tendons becomes tied more strongly, so the tendon’s tension is slowly reduced until there is slack. For this reason, as shown in Figure 14, Figure 15, Figure 16, Figure 17, Figure 18 and Figure 19, applied external forces are decreasing at every cycle, and therefore do not obtain the same maximum applied force. 

The estimated *F_z_*, *M*_x_, and *M_y_* are compared with the benchmark results, shown in Figure 14, Figure 15, Figure 16, Figure 17, Figure 18 and Figure 19 and Table 2. It was observed that the maximum error *F_z_* and *M_x_* is around 1.8 N (22.5%) and 1.6 N**·**cm (17.7%), respectively, in the case of the benchmark (*F_z_*, *M_x_*) as depicted in Figure 12b(①) and Figure 15. The maximum error for *M_y_* is around 1.7 N**·**cm (16%) for physical values, (*F_z_*, *M_y_*) as depicted in Figure 12b(③) and Figure 16, and Table 2.

The results of the repeatability test are presented in Table 3, showing the capability of the force sensor in reproducing the same condition of the force, and two moments. It can be seen that the maximum *F_z_* and *M_x_* repeatability is around 2.2% and 3.7%, respectively, in the case of the benchmark force (*F_z_*, *M_x_*) as depicted in Figure 12b(①) and Figure 15. The maximum *M_y_* is around 2.9% in the case of the benchmark force (*F_z_*, *M_x_*, *M_y_*) as depicted in Figure 12b(⑧) and shown in Table 3 and Figure 19. 

The results of the hysteresis tests are shown in Table 3; an external force is applied onto the sensor from zero to the maximum value, and then released from the maximum to zero on the load fixture again. During one cycle, the sensor’s characteristic curves exhibit a considerable hysteresis as shown in Figure 14, Figure 15, Figure 16, Figure 17, Figure 18 and Figure 19. The hysteresis in the case of the benchmark forces (*F_z_*, *M_x_*) and (*F_z_*, *M_y_*) applied (Figure 12b(①,③) can be observed in Figure 15 and Figure 16, and maximum hysteresis values are shown in Table 3.

The crosstalk between the sensing elements was summarized in Table 4. When an external force is applied, e.g., *F_z_* (force along the *z*-axis), the two moments *M_x_* and *M_y_* should ideally remain zero. Similarly, if (*F_z_*, *M*_x_) is applied on the sensor, *M_y_* should ideally remain zero, and if (*F_z_*, *M_y_*) is applied on the sensor, *M_x_* should remain zero. However, the results from the crosstalk experiments show that applying maximum values of force *F_z_* and moments (*F_z_*, *M*_x_), and (*F_z_*, *M_y_*) respectively on the load fixture ⓪, ①, ②, ③, and ④, influences the other force/moment readouts, as shown in Figure 14, Figure 15, Figure 16, Figure 17 and Figure 18 and Table 4. When *F_z_*, (*F_z_*, *M_x_*), and (*F_z_*, *M_x_*) are applied on the load fixture ⓪, ①, and ③, maximum crosstalk values arise along *M_x_* of 0.078 N**·**cm/N and *M_y_* of 0.054 N**·**cm/N (Figure 14), along *M_y_* of 0.1284 N**·**cm/N and 9.26% (Figure 15), and along *M_x_* of 0.0656 N**·**cm/N and 4.84% (Figure 16) can be observed.

## 4. Discussion

The calibration matrix was calculated analytically (Equation (16)) and compared with the empirically obtained one (Equation (19)). The analytically induced Equations (7)–(13) assumed the three deflections were measured on the centre points underneath the simply-supported beam where external force is transmitted to, as shown in the Figure 6. However, in the real sensor application, the optoelectronic sensors and the mirrors were attached closely to the centre of the sensor structure as shown in Figure 1 and Figure 2, and they were not positioned exactly at the centre and exactly toward the centre of the sensor structure. For these reasons, the simulation result (Figure 10b) and the experiment result (Figure 13b) are slightly different. Moreover, due to the hysteresis, the output voltage readings of the three optoelectronic sensing elements obtained from the loading and unloading processes are not exactly the same (Figure 13). However, the calibration matrix is calculated using the entire set of voltage readings from both loading and unloading, therefore the calibration matrices are not exactly the same. 

The errors and the crosstalk are associated with hysteresis, as shown in Figure 13. Due to the hysteresis, the calculated calibration matrix causes the errors and crosstalk in values of the *F_z_*, *M_x_*, and *M_y_* to be higher. It also negatively influences the repeatability of the sensor. As shown in Figure 14, Figure 15, Figure 16, Figure 17, Figure 18 and Figure 19, it is observed that due to hysteresis, the magnitudes of the estimation forces/moments are getting larger in comparison with real forces/moments, as cycle numbers increase. This explains why the repeatability errors of *F_z_*, *M_x_*, and *M_y_* are relatively large. The static simulation result (Figure 10d–f) shows a low crosstalk of around less than 0.5% (note that the static simulation cannot show hysteresis properties). However, it is noted that the hysteresis level of the multi-axis force/torque sensor proposed in this study is higher than that seen in commercial sensors (such as ATI sensors). Hence, the use of metals in the fabrication of future sensors is planned, instead of the currently used ABS plastic materials (the sensor structure is fabricated from Visijet EX200 via a Projet HD 3000 3D production system.

**Figure 20:**
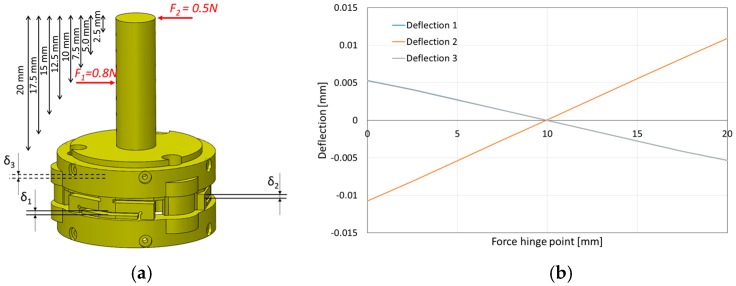
Effect of horizontal forces on the deflection of sensor beams. (**a**) Simulation of force application; (**b**) Deflections as a function of different amounts of applied moment (the resultant force is constant).

**Figure 21:**
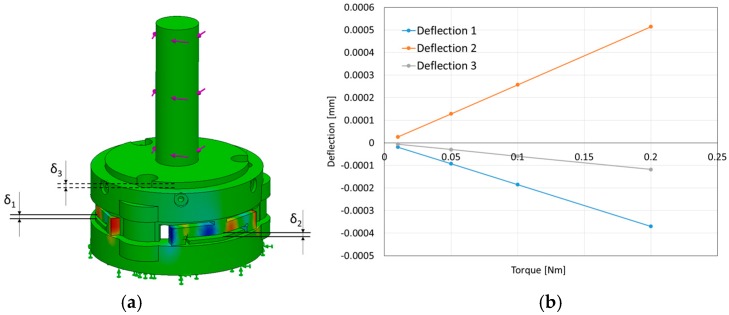
Effect of moment around the z-direction on sensor beam deflection. (a) Simulation; (b) Deflection of the beams as a function of applied moment.

Using our calibration device, *F_z_* could be applied on the sensor independent of *M_x_* and *M_y_.* However, the pure bending moments *M_x_* and *M_y_* could not be independently exerted on the sensor. Hence, in our setup, we had to apply coupled force/moment values to our sensor to calibrate it. As such, in order to guarantee that the sensor readings are not affected by forces and torques that it does not measure (i.e., forces on the *x* and *y* directions and moment around the z direction), a set of simulations were carried out that are explained in the following (Figure 20 and Figure 21). 

In the simulation, two opposing forces were applied to a shaft along the sensor’s z direction. The smaller force *F*_2_ was applied on the tip of the shaft (0.5 N), while an opposing force of higher magnitude *F*_1_ was applied along the shaft at different points (0.8 N). Since the two forces have different magnitudes, a net horizontal force was always resulted. However, the resulting moment depended on the point of application of *F*_1_. When the length of the lever arm at the point of application of *F*_1_ was such that the moment equalled the one generated by *F*_2_, the resulting moment was zero, while the resultant force was *F*_2_ − *F*_1_ = 0.3 N. It can be seen in Figure 20 that, at this point, the deflection of all three beams was negligible. Thus, this structure is not affected by these lateral forces.

Concerning the moment around the z direction, a similar simulation was carried out, which demonstrated that the sensor was also not affected noticeably by this element (*M_z_*). Figure 21 shows the deflection of the three beams in this situation. It can be seen that the deflection is negligible (in the order of 10^−7^ m, around 100 times smaller than for a moment of similar magnitude around one of the other axes).

In the future, we will propose an advanced calibration device that can apply coupled force/torque as well as pure moments. In addition, advances in optoelectronics technology have allowed photo sensors to become highly miniaturized, whilst remaining very cheap. Small-sized optoelectronics were recently achieved; an example includes the NJL5901R-2, 1.0 × 1.4 × 0.6 mm^3^, by New Japan Radio Co., Ltd. (Tokyo, Japan). We, therefore, foresee that the size of our sensor structure can be further reduced [50].

It should be mentioned that, in Section 3.1, an ATI Nano IP65 six-axis force/torque sensor, which is a standard measurement device, was used for calibration. However, this sensor is not a high precision sensor, whose inaccuracies are around 1%. According to the measurement theory, the standard measurement device should be more precise than that of the calibrated sensor. Hence, in our future studies we will either use standard weights or a more precise six-axis force/torque sensor for the purpose of calibration.

## 5. Conclusions and Future Works

In this paper, details of an optoelectronic and simply-supported beam based three-axis force/torque sensor, which can be adapted to bespoke mechanical structures for the purpose of force/torque measurements within robot systems, have been described. An example application of our force/torque sensing concept to a flexible continuum cylindrical MIS (Minimally Invasive Surgery) manipulator (the STIFF-FLOP arm) has been presented, including its design, fabrication, and evaluation tests. In addition, a strategy to obtain the calibration matrix for reliable force prediction, applying the multiple linear regression method, is proposed. Finally, the effectiveness of the proposed three-axis force sensor has been validated through a set of experiments evaluating its properties such as crosstalk, hysteresis, repeatability and error. 

Although, in this paper, we verified a sensor development method of building three-axis force/torque sensor based on optoelectronic technology and simply-supported beam, as future work, a metal structure will be considered to re-evaluate the sensor performance properties conducted in the Section 3. 

## Figures and Tables

**Table 1:** Measurable Range of Force *F_z_*, and Moments *M_x_* and *M_y_*.

Force and Moment Ranges	Sensor Structure Size, Height *H* and Diameter *D*	Sensor Structure’s Design Variables
*F_z_* ±7 N	*H* = 12 mm *D* = 24 mm	*L* = 6 mm, *L*_1*x*_ = 9.3 mm, *L*_1*y*_ = 5.4 mm, *L*_2*x*_ = 9.3 mm, *L*_2*y*_ = 5.4 mm, *L*_3*y*_ = 10.8 mm, *h* = 1.5 mm, and b = 1.5 mm
*M_x_* ±8.0 N·cm		
*M_y_* ±8.0 N·cm		

**Table 2:** Sensor Performance Property: Range and Error.

Force/Moment	Range	Maximum Error
*F_z_*	±8.0 N	1.8 N (22.5%)
*M_x_*	±9.0 N·cm	1.6 N·cm (17.7% )
*M_y_*	±11.0 N·cm	1.7 N·cm (16.0%)

**Table 3:** Sensor Performance: Repeatability and Hysteresis.

Force/Moment	Repeatability	Hysteresis
*F_z_*	2.2%	28.7 %
*M_x_*	3.7%	22.6%
*M_y_*	2.9%	21.1%

**Table 4:** Sensor performance property: Crosstalk.

	Force/Moment Applied				
	*Fz*	*F_z_ M_x_*		*F_z_ M_y_*	
Force/Moment	*M_x,y_/F_z_* (N·cm/N)	*M_y_*/*F_z_* (N·cm/N)	*M_y_*/*M_x_* (%)	*M_x_*/*F_z_* (N·cm/N)	*M_x_*/*M_y_* (%)
*F_z_*	*F_z_*	*F_z_*		*F_z_*	
*M_x_*	0.078	*M_x_*		0.0656	4.84
*M_y_*	0.054	0.1284	9.26	*M_y_*	
